# Single‐cell RNA sequencing reveals the heterogeneity and intercellular communication of hepatic stellate cells and macrophages during liver fibrosis

**DOI:** 10.1002/mco2.378

**Published:** 2023-09-17

**Authors:** Sheng Cheng, Yunhan Zou, Man Zhang, Shihao Bai, Kun Tao, Jiaoxiang Wu, Yi Shi, Yuelan Wu, Yinzhong Lu, Kunyan He, Peng Sun, Xianbin Su, Shangwei Hou, Bo Han

**Affiliations:** ^1^ Department of General Surgery Tongren Hospital Shanghai Jiao Tong University School of Medicine Shanghai China; ^2^ Key Laboratory for Translational Research and Innovative Therapeutics of Gastrointestinal Oncology Hongqiao International Institute of Medicine Tongren Hospital Shanghai Jiao Tong University School of Medicine Shanghai China; ^3^ Department of Biochemistry and Molecular Cell Biology Shanghai Key Laboratory for Tumor Microenvironment and Inflammation Shanghai Jiao Tong University School of Medicine Shanghai China; ^4^ Key Laboratory of Systems Biomedicine (Ministry of Education) Shanghai Center for Systems Biomedicine Shanghai Jiao Tong University Shanghai China; ^5^ Department of Pathology Tongren Hospital Shanghai Jiaotong University School of Medicine Shanghai China; ^6^ Key Laboratory for the Genetics of Developmental and Neuropsychiatric Disorders Bio‐X Institutes Shanghai Jiao Tong University Shanghai China; ^7^ eHealth Program of Shanghai Anti‐Doping Laboratory Shanghai University of Sport Shanghai China; ^8^ Department of Anesthesiology Tongren Hospital, Shanghai Jiao Tong University School of Medicine Shanghai China

**Keywords:** hepatic stellate cell, heterogeneity, intercellular crosstalk, liver fibrosis, macrophage, ScRNA‐seq

## Abstract

Uncontrolled and excessive progression of liver fibrosis is thought to be the prevalent pathophysiological cause of liver cirrhosis and hepatocellular cancer, and there are currently no effective antifibrotic therapeutic options available. Intercellular communication and cellular heterogeneity in the liver are involved in the progression of liver fibrosis, but the exact nature of the cellular phenotypic changes and patterns of interregulatory remain unclear. Here, we performed single‐cell RNA sequencing on nonparenchymal cells (NPCs) isolated from normal and fibrotic mouse livers. We identified eight main types of cells, including endothelial cells, hepatocytes, dendritic cells, B cells, natural killer/T (NK/T) cells, hepatic stellate cells (HSCs), cholangiocytes and macrophages, and revealed that macrophages and HSCs exhibit the most variance in transcriptional profile. Further analyses of HSCs and macrophage subpopulations and ligand–receptor interaction revealed a high heterogeneity characterization and tightly interregulated network of these two groups of cells in liver fibrosis. Finally, we uncovered a profibrotic Thbs1+ macrophage subcluster, which expands in mouse and human fibrotic livers, activating HSCs via PI3K/AKT/mTOR signaling pathway. Our findings decode unanticipated insights into the heterogeneity of HSCs and macrophages and their intercellular crosstalk at a single‐cell level, and may provide potential therapeutic strategies in liver fibrosis.

## Graphical abstract

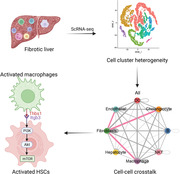

## 1 INTRODUCTION

Liver fibrosis is characterized by an excessive accumulation of extracellular matrix (ECM) components at the site of liver injury, which is considered to be the pathological foundation for the progression of other serious liver diseases such as cirrhosis and liver cancer.^1^ The malign progressive nature of liver fibrosis is the leading cause of death worldwide.^2^ The rise in hepatic fibrosis cases highlights the urgent need for improved antifibrotic therapies.

Hepatic nonparenchymal cells (NPCs), including hepatic stellate cells (HSCs), endothelial cells, cholangiocytes, and diverse immune cells are involved in the intricate regulation of liver fibrosis.^3^, ^4^, ^5^, ^6^ Their transcriptome reprogramming, heterogeneity and interhepatic crosstalk contribute to the pathogenesis of liver fibrosis. HSCs, the key type of NPCs, reside in the perisinusoidal space of Disse and are activated in response to various stimuli such as transforming growth factor‐β (Tgfβ),^7^ tumor necrosis factor alfa (TNF‐α)^8^ and platelet‐derived growth factor (PDGF).^9^, ^10^ Activated HSCs (aHSCs) are recognized as the primary source of ECM in fibrotic livers. In addition to ECM production, aHSCs can regulate ECM turnover by producing degradative enzymes (matrix metalloproteinases and metalloproteinases),^11^ promote angiogenesis by producing angiopoietin I,^12^ and mediate infiltration of immune cells into the injured livers by secreting cytokines.^8^, ^13^ These functional roles of aHSCs in liver fibrosis make targeting aHSCs a promising antifibrotic treatment. However, the wide heterogeneity of aHSCs, as revealed by recent single‐cell transcriptomic analysis,^14^, ^15^, ^16^ poses a great challenge to existing antifibrotic therapies, partly because most clinical treatments target only a subset of aHSCs. Phenotyping of HSCs at single‐cell level and uncovering the mechanisms of HSCs activation may inspire antifibrosis therapeutic approaches by targeting multiple subpopulations of aHSCs or novel HSCs activation pathways.

The cell–cell signaling network holds the richness, complexity, and spatiotemporal natures within liver physiology and disease. Interaction with other cell types in the liver is one of the essential mechanisms of HSC activation and phenotypic transition in the progression of liver fibrosis.^16^ Recent liver RNA sequencing data highlight HSCs as a crucial signaling hub for immune cell communication. Of all hepatic cell types, HSCs exhibit some of the most numerous and varied contacts, including those with macrophages and, to a lesser extent, T, B, and NKT cells.^14^, ^17^ There is now a substantial body of compelling evidence derived from experimental models that extensive cell–cell communication between hepatic immune cells and HSCs clearly regulate the progression and regression of liver fibrosis.^18^, ^19^ For instance, macrophages, perhaps the most widely studied immune cells in liver fibrosis, show duality in the fibrotic liver by expressing different immunomodulatory factors to activate or inhibit HSCs activition.^16^, ^20^, ^21^ Similarly, the balance of T cells in livers determines whether they form a profibrotic or antifibrotic network with HSCs.^22^, ^23^ In single‐cell sequencing studies, ligand–receptor signaling has emerged as a powerful tool for establishing the interhepatic crosstalk among liver cells.^14^ Unbiased ligand–receptor interaction analysis of the human liver fibrotic niche has revealed that scar‐associated macrophages induce HSCs proliferation through TNFSF12–TNFRSF12A and PDGFB–PDGFRA signaling, as well as the endothelial cells promote Notch1–JAG1‐mediated HSC activation.^15^ Despite these, whether other new candidate intercellular interaction regulatory mechanisms of HSCs activation exist remains poorly understood.

Here, we performed scRNA‐seq to examine the heterogeneity of NPCs and identified the major cell types in normal and fibrotic livers. The differential gene expression analysis revealed that HSCs and macrophages have the most altered transcriptional profile. Furthermore, we uncovered the subpopulations of HSCs and macrophages and the molecular details of their heterogeneity. We also constructed the interhepatic crosstalk between HSCs and other liver cell types, which paired with Thbs1 treatment and coculture analyses revealed that the profibrotic Thbs+ macrophages subcluster aHSCs via PI3K/AKT/mTOR signaling pathway. The current study validates that the heterogeneity of HSCs and macrophages and their interaction link to the complicated pathology of liver fibrosis, which provides a new perspective for exploring targeted antifibrotic therapies.

## 2 RESULTS

### 2.1 Clustering of scRNA‐seq data identifies NPC phenotypes

**FIGURE 1:**
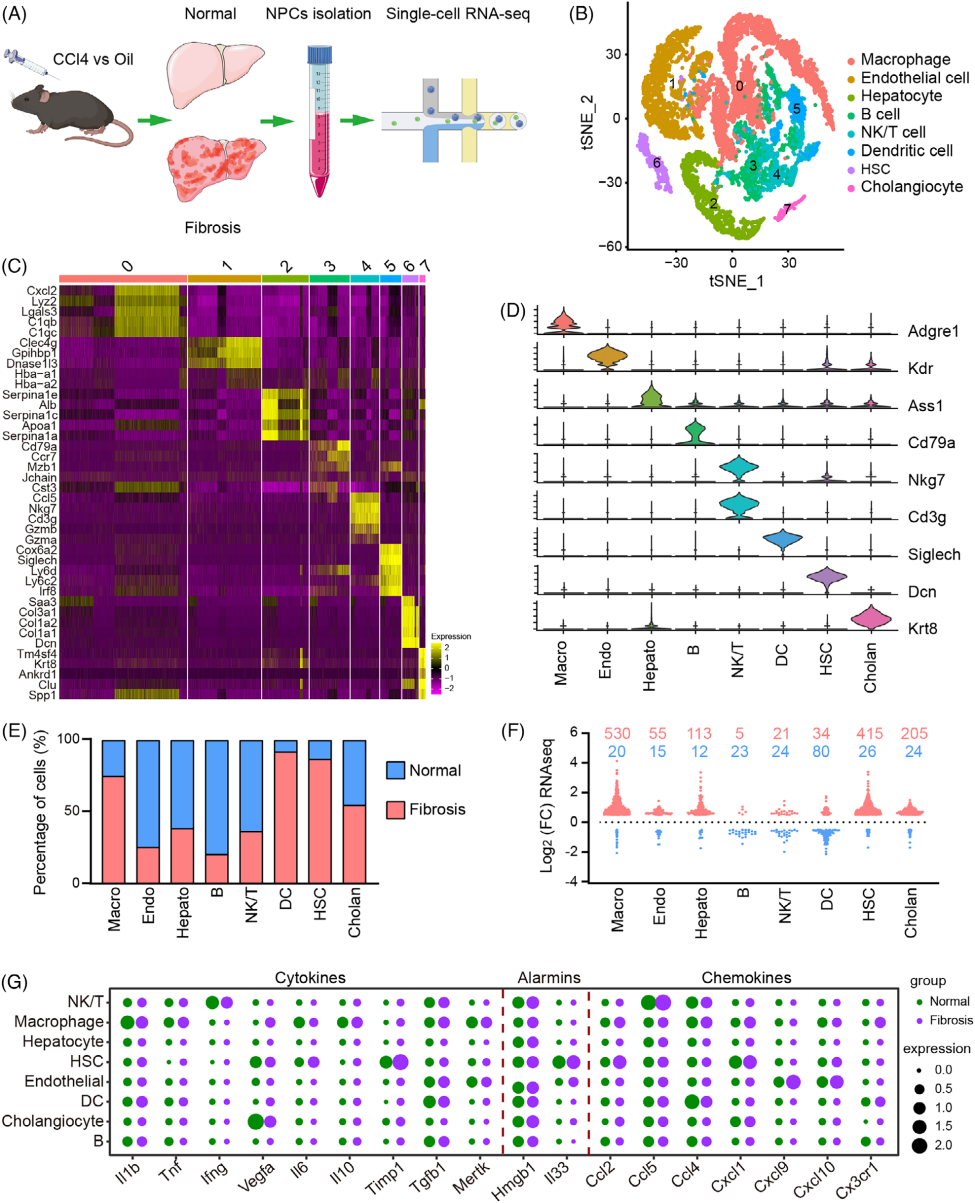
ScRNA‐seq of nonparenchymal cells (NPCs) from normal and fibrotic mouse livers. (A) Outline of the NPCs isolation and single‐cell RNA‐seq analysis. This graphical work model was generated by applying the Biorender website (https://biorender.com/). (B) t‐Distributed stochastic neighbor embedding (t‐SNE) visualization of major liver cell populations identified after unsupervised clustering. HSC, hepatic stellate cell. (C) Heatmap of cluster top five marker genes. Numbers on the top correspond to the clusters in B. (D) Violin plots showing representative enriched gene expression for each cluster. (E) Percent contribution of normal (blue) and fibrotic (red) mouse liver cells in each cluster. (F) Distribution for upregulated (red) and downregulated (blue) genes in each cluster. (D) Dot plot showing the expression of cytokines, chemokines, and alarmins in all cell populations.

In order to comprehensively define the heterogeneity of NPCs in normal and fibrotic livers, we prepared a mouse model of CCl_4_‐induced liver injury and fibrosis and harvested liver NPCs 6 weeks after the first CCl_4_ injection, with olive oil treatment as a control (Figure S1A). scRNA‐seq by the 10× chromium platform was performed on NPCs from two pairs of mice (Figure 1A). After quality control and filtering, 15,357 cells were analyzed and a median of 1603 genes per cell was measured (Figure S1B). Unsupervised clustering analysis of the control and fibrotic liver data showed eight major clusters: endothelial cells, hepatocytes, dendritic cells (DCs), B cells, natural killer/T (NK/T) cells, HSCs, cholangiocytes, and macrophages, based on expressing known markers (Figures 1B–D and S1C). By comparing our dataset with a recent scRNA‐seq study of human livers,^15^ we found that the major liver cell types shared common marker genes in mice and humans, indicating their highly conserved transcriptomic signature. The clustering analysis revealed the major cell populations between normal and fibrotic livers are similar, but the cluster size varies across fibrotic progression (Figure 1E), suggesting that fibrosis causes a changed constitutive fraction of liver cells. To assign which cell types in fibrotic livers likely contribute to transcriptome changes, we analyzed differential gene expression for each cluster in normal and fibrotic livers. As shown in Figure 1F, the macrophage and HSC‐enriched genes were primarily liver fibrosis‐induced genes, and genes specific to DC cluster were downregulated. These results demonstrated that liver fibrosis pathogenesis is associated with cell type‐specific distribution change and transcriptional dynamics. Next, to overview liver injury‐associated molecular patterns such as inflammatory cytokines, chemokines, and alarmins, which are involved in the initiation and progression of hepatic fibrosis but lack sufficient knowledge of their biological origin in the liver. For example, *Il33* is regarded as the classic hepatic alarmin and is reported to be released from the injured hepatocytes in the fibrotic liver.^24^, ^25^ Our data, however, showed that *Il33* expression was restricted to HSCs, underscoring the critical role of HSC in response to liver injury/fibrosis (Figure 1G). In the following analysis, we first delineated the changes in the proportion and transcriptional profile of HSC to provide insights into the role of HSC in liver fibrosis at the single‐cell level.

### 2.2 Identification of the genetic changes in aHSCs

**FIGURE 2:**
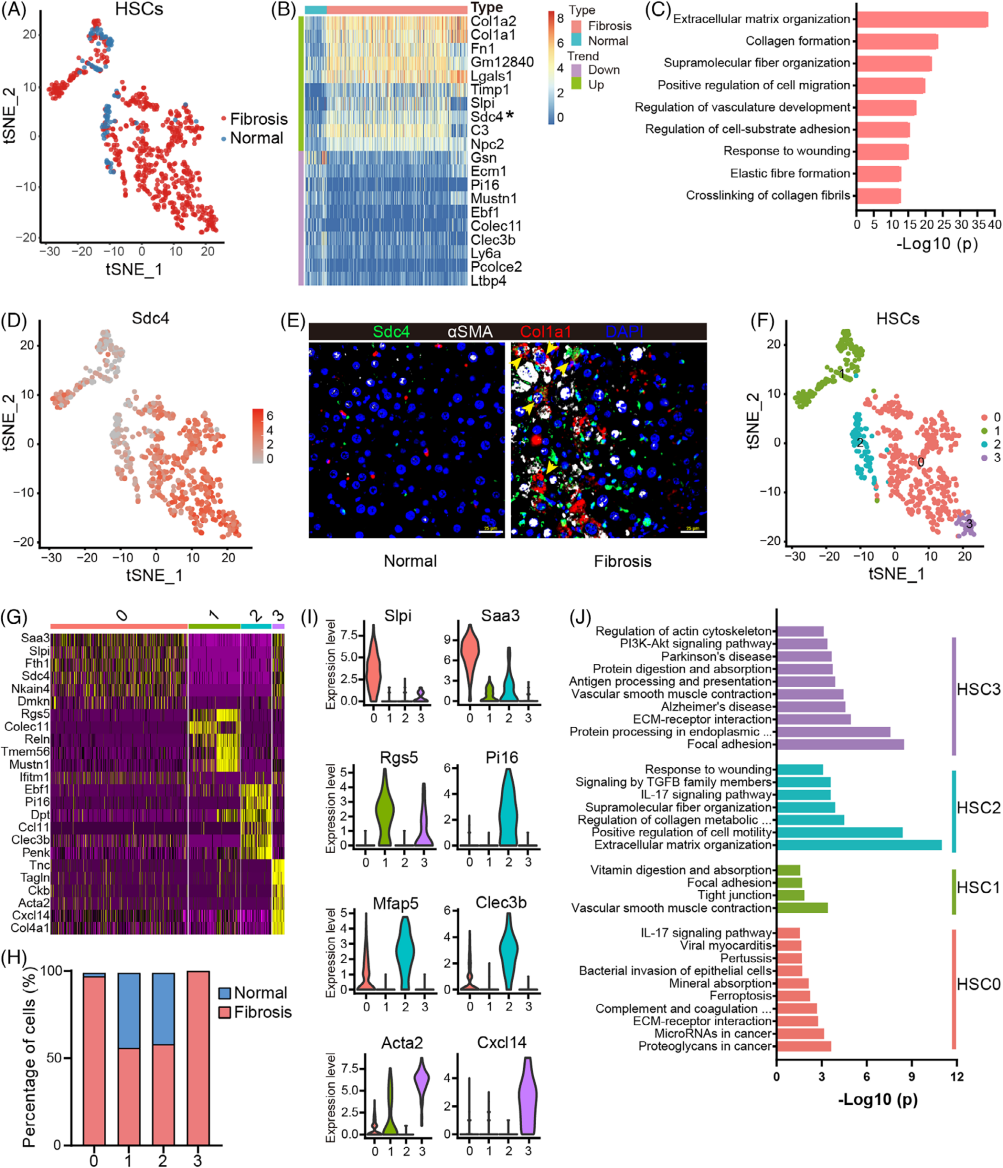
Identification of hepatic stellate cell population. (A) t‐SNE showing the clustering of all HSCs from normal and fibrotic mouse livers. (B) Heatmap showing the expression levels of DEGs in HSCs of the fibrotic mice relative to normal mice. Asterisk indicates the differentially expressed *Sdc4* gene. (C) Representative GO terms enriched with DEGs in HSCs from fibrotic mice. (D) t‐SNE visualization of the cellular expression of *Sdc4* genes in HSC cluster. (E) Immunofluorescence for Sdc4 in normal and fibrotic livers (scale bar, 25 μm). (F and G) t‐SNE showing the second‐level clustering of HSCs and heatmap showing the top six marker genes in four HSCs subclusters. (H) Percent contribution of normal (blue) and fibrotic (red) HSCs in each subcluster. (I) Violin plots showing the expression of representative marker genes of all four subclusters. (J) Representative GO terms enriched with DEGs in four subclusters of HSCs.

aHSCs have been recognized as the most critical contributor to the development of liver fibrosis.^26^ Our RNA‐sequencing analysis revealed that CCl_4_ treatment significantly increased the proportion of these HSCs (Figures 2A and S2A), which is consistent with the finding that those aHSCs will differentiate into highly proliferative and migrating myofibroblasts that accumulate in areas of hepatocyte necrosis and apoptosis in response to liver injury.^27^ Next, to identify cell‐type‐specific alterations in gene expression induced by CCl_4_, we used single‐cell differential expression software to detect differentially expressed genes (DEGs) from single‐cell data. We discovered that the CCl_4_‐induced DEGs were particularly concentrated in pathways for ECM organization, collagen fibril formation, cell migration, and wound healing (Figures 2B and C). All those pathways were associated with liver injury and ECM deposition, two main phenotypes of liver fibrosis. The top 10 genes that were highly expressed in CCl_4_‐treated HSCs are well‐established aHSCs markers including collagens (*Col1a1* and *Col1a2*), fibronectin (*Fn1*), and metalloproteinase inhibitor 1 (*Timp1*),^28^ reflecting activation of the fibrosis gene program.

Interestingly, aortic smooth muscle actin (αSMA/*Acta2*), which is broadly accepted as a classical aHSCs marker,^29^ was only expressed centrally in a subset cluster of HSCs in fibrotic mouse models, suggesting that αSMA is not the universal identifier for all aHSCs. Therefore, we sought to identify the new markers that are uniquely and uniformly expressed on all aHSCs. By examining the upregulated DE genes, we found that Syndecan‐4 (*Sdc4*), one of the top 10 genes was highly upregulated on aHSCs, but barely expressed on resting HSCs (Figure 2D). Using immunofluorescence costaining for αSMA, Col1a1, and Sdc4, we confirmed the presence of triple‐positive cells in fibrotic livers (Figure 2E).

Several single‐cell sequencing analysis have reported the heterogeneity of aHSCs.^30^, ^31^, ^32^ We used a similar unsupervised approach as described above to discover HSCs subtypes and found that they can be separated into four different subclusters, which we termed HSC0 to HSC3 (Figure 2F). HSC0 was almost aHSCs and can be defined by the high expression of the inflammation associated *Slpi*, *Saa3*, and *Cxcl5* (Figures 2G–I and S2B). Functional enrichment analysis of these upregulated genes suggested that HSC0 may be a group of immunomodulatory aHSCs subpopulations involved in the immune response during liver fibrosis, but not in the regulation of cell proliferation (Figure 2J). HSC0, with myeloid leukocyte characteristics, suggests it is a class of myeloid myofibroblasts as previously reported.^31^ Cells in HSC1 belong to the resting HSCs as indicated by the expression of quiescence markers (e.g., *Lrat*, *Reln*, and *Rgs5*) and they are mainly from healthy livers. HSC2 includes resting and aHSCs and shows the highest expression of *Mmp2* and *Mmp3*, microfibril‐associated glycoproteins (*Mfap4* and *Mfap5*), *Tnxb* and *Dpt* (Figures 2G–I and S2B). We also found some significant marker genes of portal fibroblasts in this cluster, such as *Pi16* ^33^ and *Fbln1*,^34^ which confers a mixed phenotype characteristic of HSC2 (Figures 2I and S2B). Thus, we named HSC2 as Clec3b+ HSCs according to *Clec3b* as the cluster‐specific genes. The topmost upregulated genes in HSC2 are associated with supramolecular fiber organization and ECM building in the GO category (Figure 2J). HSC3 demonstrated the expression of cell mobility and contractility‐associated genes, ECM deposition and collagen biosynthesis genes, such as *Acta2*, *Spp1*, *Tagln*, *Cxcl14*, and various collagens (Figures 2G–I and S2B). The high expression of *Acta2* indicates that HSC3 is a population of classical myofibroblasts.^32^ Thus, our subcluster analysis revealed the presence of at least four phenotypes of HSCs during liver fibrosis, namely myeloid myofibroblasts (HSC0), resting HSCs (HSC1), Clec3b+ HSCs (HSC2), and myofibroblasts with high expression of Acta2 (HSC3).

### 2.3 The HSC ligand–receptor interaction

**FIGURE 3:**
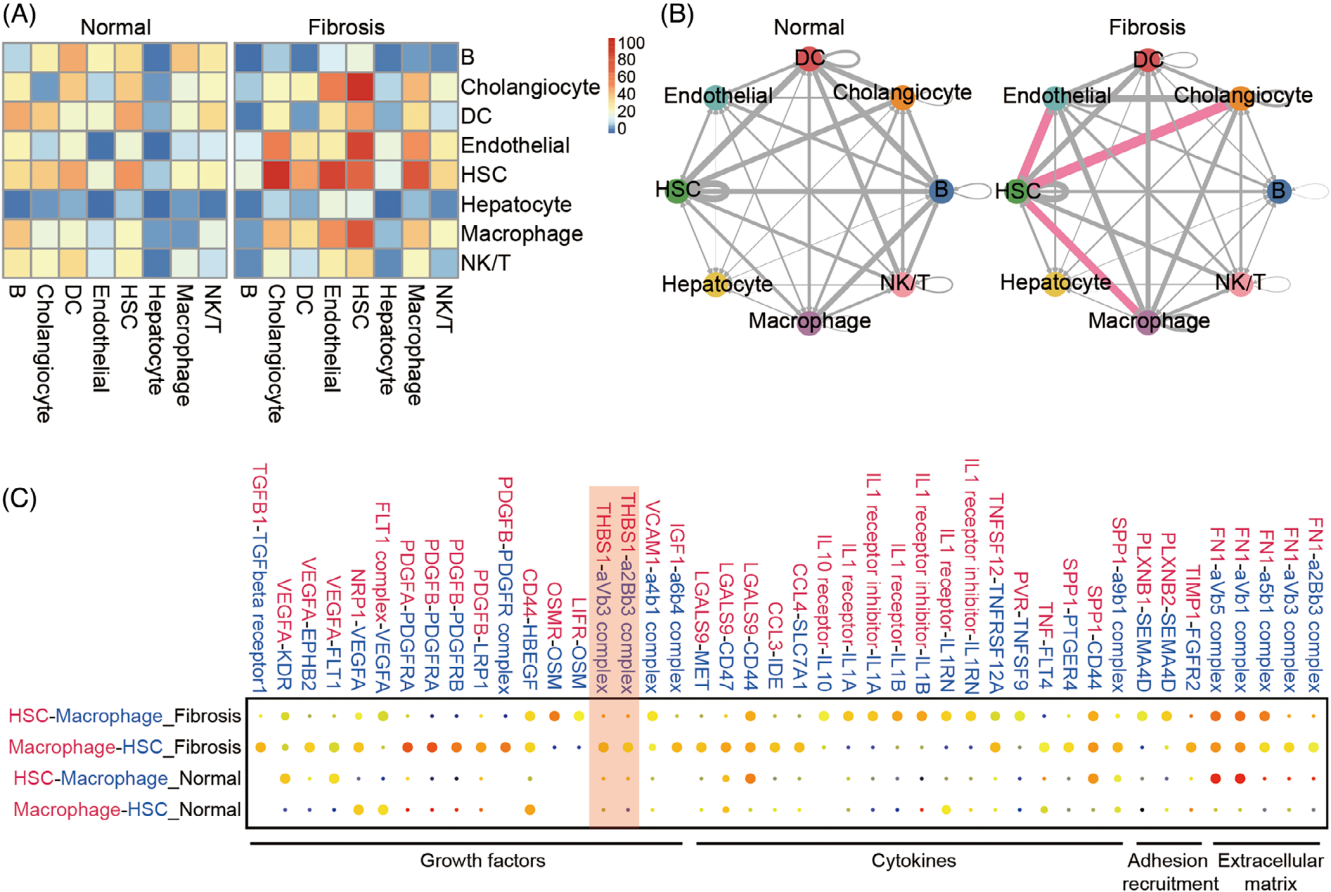
Network of cell–cell interactions in normal and fibrotic livers. (A) Heatmap showing the crosstalk between mouse hepatic cell types. Color bars represent the number of ligand–receptor pairs. (B) Capacity for intercellular communication between mouse hepatic cell types. The line thickness is proportional to the number of ligand–receptor pairs. The pink lines highlight the top three interactions with large number of ligand–receptor pairs. Loops indicate autocrine circuits. (C) Dot plot of selected ligand–receptor interactions between HSCs and macrophages in normal and fibrotic livers. The ligand (red) and receptor (blue) colors correspond to the color of the cell population that expresses them. The red rectangle indicates the interaction of Thbs1, highly expressed in fibrotic liver macrophages, with its receptors expressed in HSCs.

HSCs activation and liver fibrosis are complex processes involving the crosstalk of HSCs with other cell types in the liver.^3^, ^35^ To determine the tightly coordinated cellular response to liver fibrosis or HSCs activation, we quantified potential cell–cell interactions between all cell types in the normal liver microenvironment and fibrotic niche using a published ligand–receptor dataset.^36^ We projected those ligands and receptors in a graphical representation of all their interactions within all cell types to highlight their separation into cell‐type related modules. The numbers of the ligand–receptor pairs for each interpopulation link were counted and HSCs were identified as the most trophic cell population with dense connections to other cell types in normal and fibrotic livers (Figures 3A and B). The interaction network and heatmaps revealed a significant increase in the number of ligand–receptor pairs between HSC and macrophage, endothelial cell, and cholangiocyte in the fibrotic liver compared with the healthy one, which is in line with the claim that these cell types are involved in controlling the phenotype of HSCs and fostering the development of the liver fibrotic niche.^15^ In particular, the critical role of the crosstalk between HSC and macrophages in the progression of fibrosis has been well documented by numerous studies.^16^, ^37^, ^38^ In the fibrotic liver, macrophages consistently localize near aHSCs in areas of scar tissue. To elucidate the molecular processes by which HSCs and macrophages reciprocally regulate their phenotypes at the single‐cell level, we next used CellPhoneDB method^39^ to identify the expression of macrophage ligands and receptors and forecast its interactions with HSCs. As shown in Figure 3C, compared with the normal group, the macrophages in the liver fibrotic group secreted plenty of growth factors including *Pdgf*, *Igf‐1*, *Tgfβ1*, *Vegf*, *Timp1*, and *Osm*, suggesting that the macrophages have a promoting effect for the fibroblast proliferation. We also observed that the macrophages in the liver fibrotic niche expressed the proinflammatory cytokines, such as *Tnf*, *Il1* family, *Lgals9*, *Ccl3*, and *Tnftnsf12*, signaling to cognate receptors on fibroblasts, which indicated that macrophages can induce the inflammatory activation of HSC. In addition to growth factors and chemokines, we observed many interactions related to adhesion recruitment and ECM remodeling. The macrophages can mediate the adhesion and communication with activated fibroblasts by secreting adhesion factors *Sema4d* and *Fn1* in fibrotic models. On the other hand, the interplay results indicated that both the macrophages and aHSCs produced cytokines such as *Spp1* and *Tnfsfl12*, as well as ECM‐related *Fn1*, thereby promoting macrophage infiltration, ECM deposition, HSC activation and fibrosis.^16^ These observations support that both HSCs and macrophages are involved in regulating liver fibrosis and the bidirectional and complex functional regulation between macrophages and HSCs is the key mechanism of fibrosis progression. Therefore, the impact of macrophages on liver fibrosis requires further study at the single‐cell level.

### 2.4 Characterization of macrophage subpopulations and profibrogenic phenotype of MDMs

**FIGURE 4:**
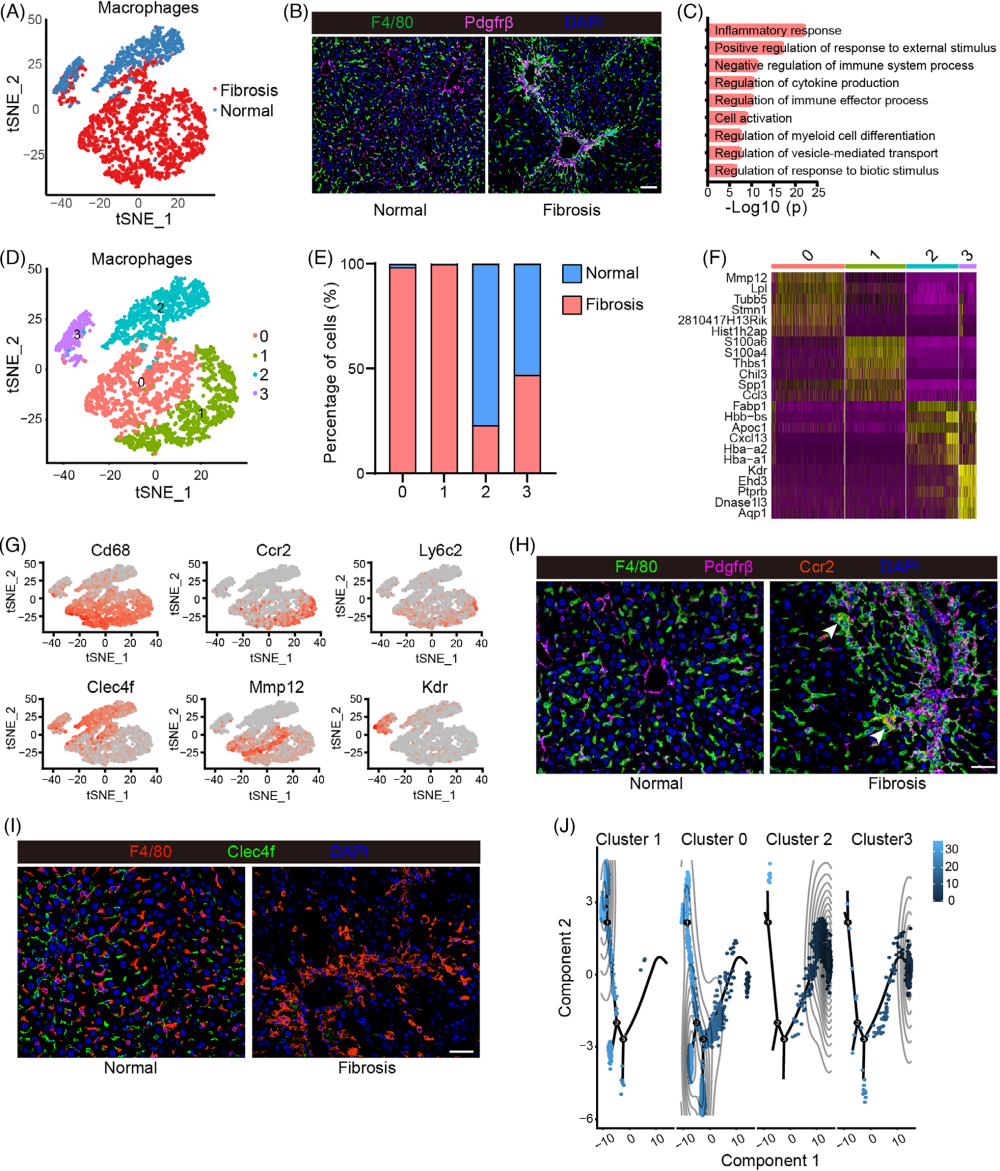
Identifying fibrosis‐associated macrophage subpopulation. (A) t‐SNE showing the clustering of all macrophages from normal and fibrotic mouse livers. (B) Immunofluorescence costaining for platelet‐derived growth factor receptor beta (Pdgfrβ) and F4/80 in normal and fibrotic mouse livers (scale bar, 50 μm). (C) Representative GO terms enriched with marker genes in macrophages. (D and E) t‐SNE shows the second‐level clustering of macrophages and the percentage contribution of normal (blue) and fibrotic (red) macrophages in each subcluster. (F) Heatmap showing the top six marker genes in four macrophage subclusters. (G) t‐SNE showing the representative marker genes of all four subclusters. (H) Immunofluorescence costaining for Pdgfrβ, F4/80, and Ccr2 in normal and fibrotic mouse livers (scale bar, 25 μm). (I) Immunofluorescence costaining for F4/80 and Clec4f in normal and fibrotic mouse livers (scale bar, 25 μm). (J) Pseudotime analysis of macrophage state transition from cluster0 to cluster4 with Monocle. The color indicates inferred pseudotime.

Evidence has shown that the macrophage expansion at fibrotic locations plays a significant role in rodent models and liver fibrosis patients.^40^, ^41^ Consistently, the phenomenon was observed in CCl_4_‐induced fibrosis models in our study. The hepatic macrophage proportion significantly increased in the liver fibrosis model compared to the control group (Figure 4A). Our immunofluorescence staining of mouse liver sections also showed a large number of macrophages aggregated in the fibrotic niche (Figure 4B). A total of 1848 DE genes were identified in the macrophages of fibrotic models. Functional enrichment analysis indicated that these upregulated genes primarily enriched in inflammatory response, immune process regulation, cell activation, and cytokine production, suggesting that the CCl_4_‐treated macrophages possessed the immune and inflammatory response signature (Figure 4C). The functional diversity of these cells may arise from their heterogeneity in the context of liver fibrosis. In order to accommodate the broad spectrum of fibrotic hepatic macrophage function and phenotypes, we further performed second‐level clustering for the macrophages. Four subclusters were generated (Mac0, Mac1, Mac2, and Mac3) and each subcluster is defined by a unique set of DEGs (Figures 4D–G and S3A). Mac0 and Mac1 were characterized by enriched expression of *Cd68*, *Itgam*, *Cx3cr1*, *Ccr2*, and *Csf1r*, suggesting that they are monocyte‐derived macrophages (MDMs) (Figures 4F, G, and S3A). Cell population source analysis and immunofluorescence labeling showed that the mouse fibrosis model contained highly expressing *Ccr2* and *Cd68* cell populations (Figures 4E, H, and S3B). Cells in cluster 2 were identified as Kupffer cells (KCs) because of the high expression of KC marker genes *Clec4f*, *Macro* and *Timd4* ^42^ (Figures 4F and G). Percent cell population analysis and Clec4f immunofluorescence staining indicated that this cell population was mainly derived from normal mouse liver (Figures 4E and I). Interestingly, the cells in cluster 3, the smallest cell cluster, expressed canonical macrophage markers such as *Clec4f*, *Adgre1* (F4/80), and *Vsig4*, but also displayed liver sinusoidal endothelial cells (LSECs) markers such as *Kdr* and *Ehd3*, suggesting that these cell populations could be the controversial macrophage‐LSEC cells that have been recently generated debate^43^, ^44^, ^45^ (Figures 4F, G, and S3A). More evidence is still required for the function and categorization of this population of cells.

Especially, cells in Mac0 captured the mature MDM character because of the downregulated expression of *Ccr2* and *Ly6c* and the upregulated expression of *Cx3cr1* ^35^ (Figures 4G and S3A). These mature MDMs highly expressed *Mmp12* and *Mmp13*, which give these macrophages the antifibrotic phenotype by degrading the ECM to promote the injured liver repair^41^ (Figures 4G and S3A). Thus, the unique gene expression profile confers a repairing effect on the injured liver by this cluster of macrophages. Unlike Mac0, cells in Mac1 are infiltrating Ly6c^high^ MDMs featured with highly expressed *Ccr2*, *Cx3cr1*, and *Itgam* (Figures 4G and S3A). Those infiltrated Ly6c^high^ monocytes express multiple cytokines and chemokines in the early phase of hepatic injury to perpetuate inflammation, but eventually, they mature into the Ly6c^low^ MDMs.^35^, ^46^ Our study mapped the differentiation from highly expressed Ly6c monocytes (Mac1) to lowly expressed Ly6c monocytes (Mac0) with pseudotemporal trajectory as shown in Figure 4J. Therefore, these cells in Mac1 are considered as early infiltrated Ly6c^high^ monocytes. By stimulating proinflammatory cascades by inflammatory gene expressing and profibrogenic cytokine releasing including *Il1b*, *Tnf*, *Lgals3*, *S100a6*, *S100a4*, *S100a11*, and *S100a10*, Mac1 was characterized as one of the profibrotic contributors during the liver fibrosis progression (Figure S3A). Unlike the monocytes infiltrating the liver during fibrosis, KCs are resident hepatic macrophages and frequently renew themselves dependent on the level of growth factors GM‐CSF (granulocyte‐macrophage colony‐stimulating factor) and M‐CSF (macrophage colony‐stimulating factor).^47^ KCs are characterized by high plasticity in response to the hepatic microenvironment changes.^48^ To examine the molecular properties of KCs, we investigated the expression of the three profibrogenic genes (*Cd163*, *CCl24* and *Cxcl13*)^49^, ^50^, ^51^, ^52^, ^53^ and two antifibrogenic genes (*Il10* and *Il4*)^54^ in Mac2 cluster and found that these profibrogenic genes were highly and specifically expressed but the antifibrogenic genes were absent or only moderately expressed (Figure S3A). Our results are consistent with observations from previous in vitro and in vivo studies that identified the profibrotic function and sentinel role of KCs in response to various environmental stimuli.^23^ However, the fate of this population may change with the progression of liver fibrosis, and experimental evidence assigning the antifibrogenic functions remains to be deciphered.

### 2.5 MDMs and HSCs interaction in fibrotic liver

**FIGURE 5:**
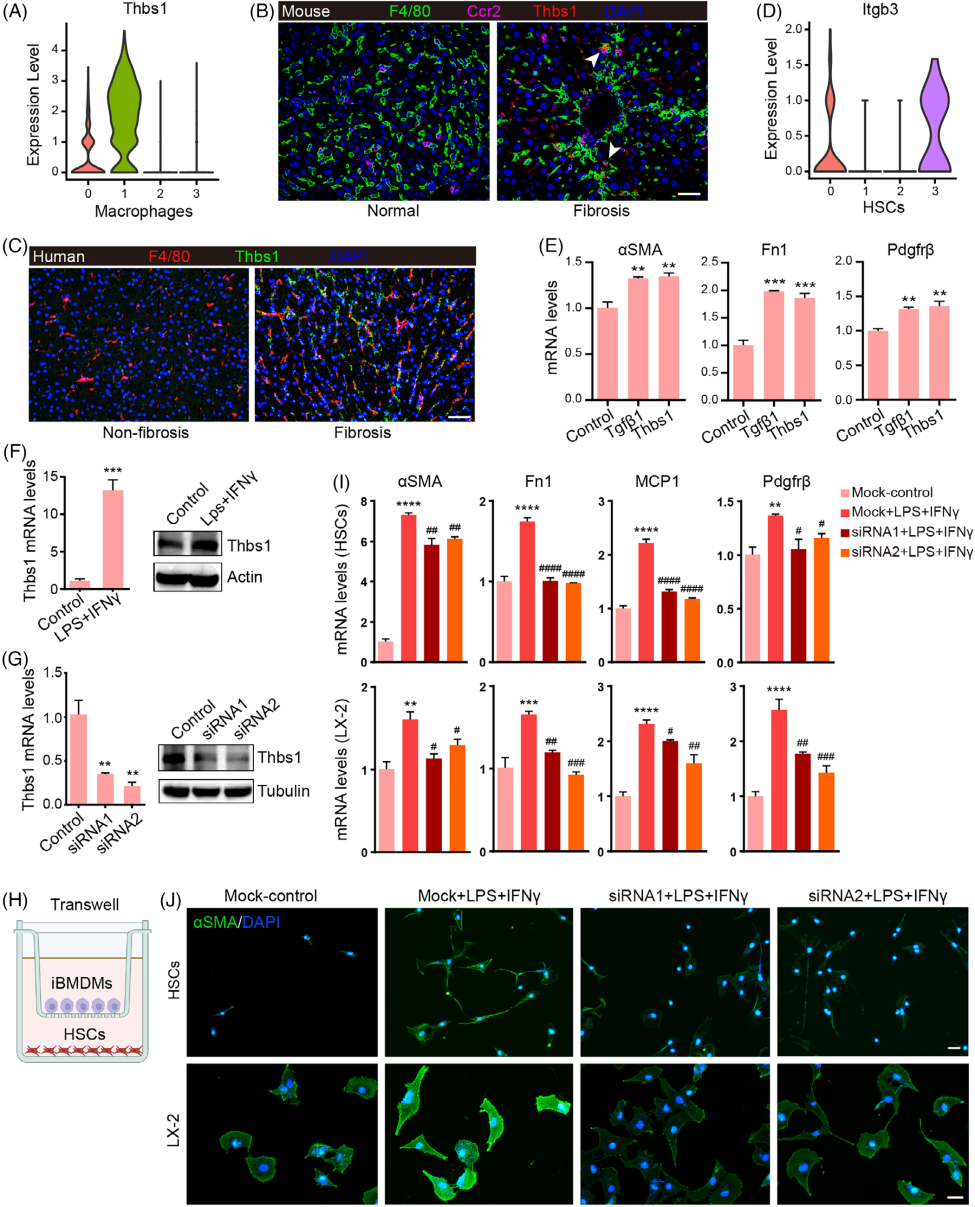
Characterization of the cellular interaction between Thbs+ macrophages and HSCs. (A) Violin plot indicates the expression of Thbs1 in macrophage subclusters. (B) Immunofluorescence costaining for F4/80, Ccr2, and Thbs1 in normal and fibrotic mouse livers (scale bar, 25 μm). (C) Immunofluorescence staining for F4/80 and Thbs1 in normal and fibrotic human livers (scale bar, 50 μm). (D) The violin plot shows the expression of Itgb3 receptor of Thbs1 in HSC subclusters. (E) Normalized mRNA expression of *αSMA*, *Fn1*, and *Pdgfrβ* in LX‐2 cells treated with Thbs1 and Tgfβ1. (F) Analysis of mRNA (left) and protein (right) expression levels of Thbs1 in iBMDMs after LPS and IFN‐γ treatment. (G) Validation of knockdown efficacy of Thbs1‐siRNA in iBMDMs by detecting mRNA (left) and protein expression (right). (H) A schematic representation showing the design of the coculture assay. This schematic representation was generated by applying the Biorender website (https://biorender.com/). (I) Normalized mRNA expression of *αSMA*, *Fn1*, *MCP1*, and *Pdgfrβ* in mouse primary HSCs and LX‐2 cells treated with Thbs1 siRNA, LPS, and IFN‐γ alone or combined (*p compared with control group, ^#^ *p* compared with LPS and IFN‐γ treatment group). (J) Representative immunofluorescence staining for αSMA in mouse primary HSCs and LX‐2 cells treated with Thbs1 siRNA, LPS, and IFN‐γ alone or combined (scale bar, 50 μm).

We further analyzed MDMs heterogeneity and observed two different MDM populations marked by low and high Thbs1 mRNA expression. Thbs1, called TSP1 (thrombospondin 1), is a multifunctional matricellular glycoprotein secreted by many cell types. Thbs1 has been implicated in the regulation of latent Tgfβ activation and macrophage‐derived Thbs1 has been proven to promote the development of nonalcoholic fatty liver disease/nonalcoholic steatohepatitis (NAFLD/NASH).^55^, ^56^ Notably, mRNA expression analysis and triple immunofluorescence staining have demonstrated that Thbs1 is specifically expressed in the profibrogenic Mac1 cluster in mouse and human fibrotic livers (Figures 4D and 5A–C), implying that Thbs1 may play a critical role in compromising liver fibrosis. By retracing the interaction between macrophages and HSC, we found that Thbs1 was derived from macrophages in the fibrosis model, and communicated with HSCs by interacting with the aVb3 (Itgb3) and a2Bb3 (Itga2b&Itgb3) complex receptors (Figure 3C). Expression analysis revealed that Itgb3 receptor was labeled only on two myofibroblast cell populations, HSC0 and HSC3 (Figures 2F and 5D), suggesting Thbs1 may promote HSC activation via ligand–receptor signaling. To confirm whether Thbs1 can activate HSCs, we examined the mRNA expression of markers of aHSCs in the Thbs1‐treated LX‐2 cell line, a partially activated human HSC line that Tgfβ1 can fully activate.^57^ Consistent with the single‐cell analysis, we observed the marked expression of *αSMA*, *Fn1*, and *Pdgfrβ* on these Thbs1‐treated LX‐2 cells (Figure 5E). In parallel, macrophages and HSCs coculture demonstrates that proinflammatory immortalized bone marrow‐derived macrophages (iBMDMs) activated by lipopolysaccharide (LPS) and interferon‐γ (IFN‐γ) upregulate Thbs1 expression and promote activation of mouse primary HSCs and LX‐2 cells (Figures 5F–J and S4A, B). Knockdown of Thbs1 in iBMDM inhibits the activating effect of polarized iBMDM on HSC (Figures 5G–J). These results illustrate that liver MDMs can induce HSC activation and a fibrotic phenotype by producing the Thbs1 mediator.

### 2.6 Thbs1 induces HSC activation through PI3K/AKT/mTOR signaling pathway

**FIGURE 6:**
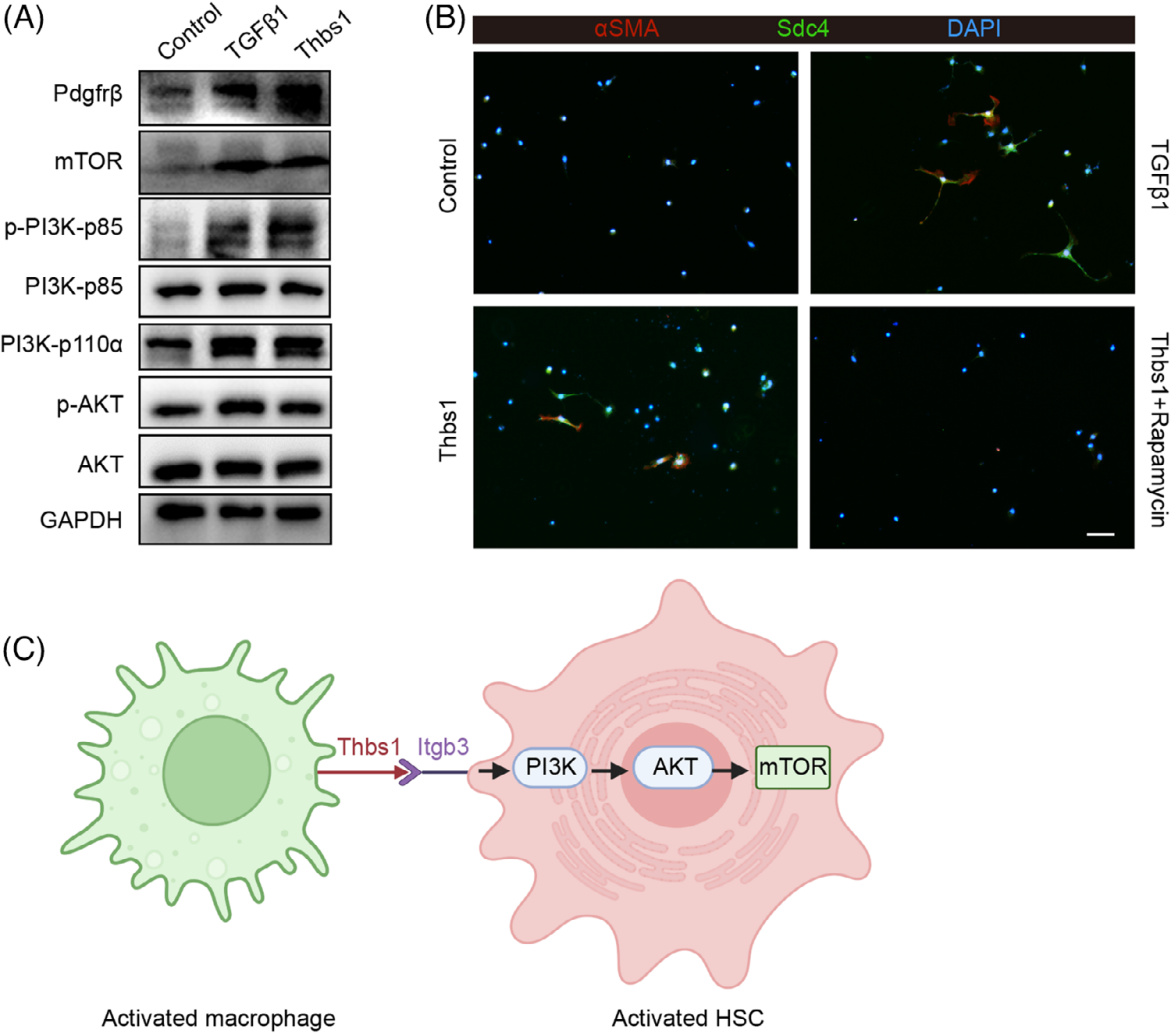
Thbs1 exerts profibrotic effects by activating the PI3K/AKT/mTOR signaling pathway. (A) Western blots show total and/or phosphorylated protein levels in primary hepatic stellate cells (HSCs) treated by Tgfβ1 and Thbs1. (B) Representative immunofluorescence staining for αSMA and Sdc4 in mouse primary HSCs treated with Tgfβ1 and Thbs1 alone and Thbs1 and rapamycin combined (scale bar, 100 μm). (C) The working model indicates that the profibrotic macrophage subpopulations can crosstalk with HSCs through the mediation of Thbs1 and activate HSCs through PI3K/AKT/mTOR signaling pathway. This graphical work model was generated by applying the Biorender website (https://biorender.com/).

To illustrate the potential molecular mechanisms underlying the action of Thbs1 on HSC activation, we performed a pathway analysis of the DE genes of the whole HSC cluster and aHSC subcluster (HSC3) (Figure 2J). The results indicated that multiple signaling pathways, including the PI3K/AKT pathway were involved in regulating HSC activation in vivo. Therefore, we hypothesized that Thbs1 contributes to the HSC activation and profibrotic effect by perturbing this PI3K/AKT pathway. Western blot results showed that thbs1 treatment could promote the expression of Pdgfrβ, PI3K‐p110α, phospho‐PI3K‐p85, phospho‐AKT, and mTOR in mouse primary HSCs (Figure 6A). Remarkably, pharmacological inhibition of mTOR activation essentially abolished the induction of activation of HSCs in response to Thbs1. By using an immunofluorescence staining analysis, we discovered that rapamycin (a particular mTOR inhibitor) fully prevented the Thbs1‐stimulated expression of a subset of aHSCs markers, including aSMA, SDC4 that we have identified in the HSC cluster, and Pdgfrβ, as well as the distinctive astral‐like morphology of aHSCs (Figures 6B and S5). These results provide strong evidence that Thbs1 induced HSC activation via PI3K/AKT/mTOR signaling pathway (Figure 6C).

## 3 DISCUSSION

Liver fibrosis has been considered to be the common pathological basis of liver cirrhosis and hepatocellular carcinoma (HCC), yet no effective antifibrotic treatment options are available. Liver cell heterogeneity and intercellular crosstalk play a vital role in liver fibrosis. However, the precise nature of cellular phenotypic transitions and interregulatory patterns remains obscure due to the limited cellular resolution of bulk transcriptomic analyses. In this study, we identify the heterogeneity of HSCs and macrophages with the most variable transcriptional profile in the fibrotic liver and construct their crosstalk using single‐cell transcriptome analysis. We also uncover the mechanism by which a subset Thbs1‐highly expressing macrophages activate HSCs.

Resting HSC to aHSC transition is a crucial event during liver fibrogenesis. aHSC harbor abnormal proliferation, activation, migration and collagen production features.^23^ In line with previous findings, our scRNA‐seq analysis data consistently found a marked expansion of HSC cluster and showed that aHSCs profoundly upregulate activation and ECM‐associated genes such as αSMA (*Acta2*) and genes encoding collagens. Although almost all aHSCs upregulated collagen production, only certain cells expressed the classical HSC activation marker αSMA, suggesting that these aHSCs are heterogeneous and that αSMA may not be a universal marker for all aHSCs. One notable advantage of scRNA‐seq analysis is its accuracy in discovering new cell type‐specific marker genes. For instance, we identified Sdc4 as a potential novel marker of aHSCs, which can be confirmed using triple immunofluorescence staining in vivo. The function of Sdc4 in aHSC is rarely studied. The available studies suggest that Sdc4 is associated with aHSC migration, a typical feature of aHSC.^58^ Further elucidation of the function of Sdc4 in aHSC will make it a new marker for aHSC identification or a potential therapeutic target for liver fibrosis. Likewise, a better understanding of the subpopulations of HSC will help to identify novel targets for antifibrotic therapy due to the different contributions of transcriptional heterogeneity of HSC to liver fibrosis. Our scRNA‐seq data identified at least four clusters of HSC in the normal and fibrotic livers. Except for the resting HSC (cluster HSC1), the remaining three HSC subsets are almost entirely derived from the fibrotic livers. Cluster HSC3 belongs to the typical myofibroblasts expressing the established myofibroblast markers, such as *Acta2*, *Spp1*, and various collagens. This population of cells has been shown to be directly associated with CCl_4_‐induced liver fibrosis in other HSC heterogeneity studies.^31^, ^32^ Although functional enrichment analysis shows that cluster HSC2 is similar to HSC3 in fiber organization and ECM remodeling, HSC2 does not express typical myofibroblast markers *Acta2* and some fibrillary collagens, instead expressing the universal fibroblast markers such as *Pi16* and *Dpt*. A recent study showed that Pi16^+^Dpt^+^ cells exhibit the capacity to serve as resource cells that can develop into activated fibroblasts or specialized fibroblasts in steady‐state mouse tissues.^30^ In light of this, HSC2 may be in an intermediate activated phenotype, acting as a pool that gives rise to the aHSCs in the livers in response to the damage brought on by CCl_4_. aHSCs mainly populate cluster HSC0, but it differs markedly from HSC2 and HSC3, as it is dominated by genes involved in immune response. The markers expressed in HSC0 include *Slpi*, *Saa3*, *Cxcl5*, and *Clec2d*, which confer immune and inflammatory properties to this population of cells. In fact, an unintended immune property of aHSCs is their role as innate immune cells mediating a range of immunomodulatory effects.^23^ A similar subcluster has been described in previous HSC heterogeneity studies,^31^ which is proposed to be associated with the development of HCC.^32^ The unanticipated heterogeneity of HSC as revealed by our scRNA‐seq and others provides new inspiration for the current treatment of liver fibrosis therapeutic strategy that targets diverse subsets of HSC.

HSCs can be activated by various profibrogenic stimuli derived from other liver cells via intercellular crosstalk. Deconstructing the interhepatic crosstalk in homeostasis and disease could provide a foundation to better understand the regulation of HSC activation in liver fibrosis initiation and progression. Ligand–receptor linkage analysis of the HSC signaling network highlights macrophages, endothelial cells, and cholangiocytes as the main cellular targets of HSC. These findings are in line with the capacity of HSC to regulate vascular and immune systems in addition to its ECM‐producing functions.^8^, ^12^, ^13^ We next focused on the interplay between HSCs and macrophages, as the inflammatory activity of liver macrophages dominates during HSC activation. Using CellPhoneDB method, we observed that the macrophages in fibrotic livers secret growth factors, profibrotic chemokines and adhesion factors to mediate the proliferation, activation, and ECM remodeling of HSCs. Activation of HSC is controlled by these complex immunologic interactions implying the sensational role of macrophages in liver fibrosis. However, several lines of evidence have demonstrated the functional complexity and heterogeneity of macrophages in fibrotic livers, and exactly which specific subgroups of macrophages contribute to HSC activation through these interacting pathways requires an in‐depth analysis of macrophage clusters at the single‐cell level.

Our single‐cell analysis revealed four subsets of macrophages under normal and fibrotic livers, including KCs (cluster Mac2), macrophage‐LSECs (cluster Mac3), antifibrotic MDMs (cluster Mac0), and profibrotic MDMs (cluster Mac1). Since monocyte recruitment is essential for hepatic fibrogenesis, our work has subsequently focused on characterizing the MDM populations. The unique Thbs1 expression in Mac1 made this cluster the Thbs1+ highly expressed MDM subpopulation. Our immunofluorescence staining demonstrated that this population existed in the fibrotic livers of mice and people. The current studies have reported that Thbs1 has the potential to regulate HSC activation and promote NAFLD/NASH progression, suggesting that as a profibrotic gene, Thbs1 partially contributed to the profibrotic profile of cluster Mac1. Our Thbs1‐induced HSC activation experiments, profibrotic iBMDMs–HSC coculture and HSC–macrophage interaction analysis suggest that Thbs1 may activate HSC by interacting with aVb3 and a2Bb3 complex receptors on HSC, but the exact regulatory mechanism remains unclear. By enriching the pathways implicated in HSC activation, we identified PI3K/AKT signaling pathway that may be involved. The PI3K/AKT signaling cascade has been shown to facilitate HSC activation and collagen synthesis in response to various proproliferative and fibrotic stimuli in earlier investigations.^59^, ^60^, ^61^ By modifying or inhibiting the PI3K/AKT/mTOR pathway, various potential inhibitors, such as some natural compounds, offer the ability to prevent HSC activation and liver fibrosis.^62^, ^63^, ^64^, ^65^, ^66^, ^67^ In our study, the exogenous addition of Thbs1 to induce mouse primary HSCs activation demonstrated that Thbs1 could activate HSC by promoting this signaling pathway. In contrast, pharmacological inhibition of this pathway eliminated the effect of Thbs1 activation of HSCs. Our data suggest that the profibrotic macrophage subpopulations can crosstalk with HSCs through the mediation of Thbs1 and activate HSCs through PI3K/AKT/mTOR signaling pathway, prevention of Thbs1+ macrophage‐mediated HSC activation and liver fibrosis might benefit from the inhibition of this pathway.

While this work identified the major cell types of liver NPCs, revealed the heterogeneity of HSCs and macrophages and elucidated the Thbs1‐mediated activation of HSCs by macrophages, several open questions remain. For instance, whether the immune and inflammatory HSCs are in a transitional state and may be causally related to the progression of liver fibrosis. It remains unclear whether there is the spatial dynamics between Thbs1+ macrophages and aHSCs during the development of liver fibrosis and how these dynamics contribute to liver tissue homeostasis. Further investigation is required to determine whether it is clinically feasible to prevent liver fibrosis by pharmacologically inhibiting the PI3K/AKT/mTOR signaling pathway.

In summary, our study enables a deeper understanding of the heterogeneity of HSCs and macrophages, their interhepatic crosstalk, and the molecular mechanism of the HSC–macrophage interplay in fibrosis. The identified subcluster of Thbs1+ macrophages that activate HSC may represent a novel profibrotic mechanism and potential liver fibrosis therapeutic intervention.

## 4 MATERIALS AND METHODS

### 4.1 Animals

C57BL/6 male mice (6–8 weeks old) were used to construct the liver fibrosis models. Animals were purchased from SPF (Beijing) Biotechnology Co., Ltd. and temporarily raised at our facility. To induce liver fibrosis, carbon tetrachloride (CCl_4_) (C805329; Macklin) was dissolved at 1:4 in olive oil and injected intraperitoneally at a dose of 1 μL/g body weight biweekly for 6 weeks. Mice were treated with the same dose of olive oil for 6 weeks as a control. The mouse studies were approved by the Animal Care and Use Committee of Shanghai Jiao Tong University (Approval No. 2021073003).

### 4.2 Human subjects

Human liver puncture biopsies were collected from patients with hepatitis B or liver fibrosis recruited from Shanghai Tongren Hospital. All subjects provided written informed consent. The study was approved by the ethics board of Shanghai Tongren Hospital (No. 2020‐035‐01). The punctured liver specimens were examined histopathologically, and fibrosis was assessed by experienced pathologists using the Ishak scoring system.^68^ Ishak F1 was diagnosed as nonfibrotic liver tissue and Ishak F2‐4 as fibrotic liver tissue.

### 4.3 Isolation of liver NPCs and scRNA‐seq analysis

Mice were anesthetized using isoflurane, and liver NPCs were isolated following a two‐step perfusion protocol as previously described.^14^ Briefly, the liver was perfused in situ with calcium‐free Hank's Balanced Salt Solution (HBSS) containing 0.2 mg/mL EDTA, then perfused with 0.5 mg/mL pronase E (1074330005; Merck) and 0.75 U/mL collagenase P (11213857001; Roche) for 10 mL each. The liver was minced and further digested with HBSS containing 0.2% collagenase P, 0.4 mg/mL pronase, and 0.1 mg/mL DNase I (R104159001; Roche) in 37°C with shaking for 20 min. The digestion was passed through 40 μm cell strainer and centrifuged at 50×*g* for 3 min to remove the majority of hepatocytes. The NPCs supernatant suspension was collected and treated with a Dead Cell Removal Kit (130‐090‐101; Miltenyi Biotec) according to the manufacturer's instructions to remove dead cells. The resulting NPCs were subjected to scRNA‐seq analysis using 10× Genomics Chromium Single‐Cell processing. Additional analysis was then performed by using the “Seurat” (v2.3.2) package for R (v3.5) (https://www.r‐project.org/).

### 4.4 Pseudo‐time trajectory analysis

The pseudo‐time trajectory of macrophages was constructed with Monocle.^69^ All data used for the trajectory analysis were extracted from the Seurat object. Use the “newCellDataSet” function to construct a Monocle “CellDataSet” object and visualize the results with the “plot_cell_trajectory” function.

### 4.5 Intrahepatic ligand–receptor signaling network and interaction

The ligand and receptor pairing dataset was obtained from Fantom5 (http://fantom.gsc.riken.jp), as previously described.^70^ Network connectivity was visualized using the Gephi graph tool (https://gephi.org). The receptor–ligand interactions between two cell types were performed by the CellPhoneDB method as described.^39^ To identify the most relevant interactions between cell types, only receptors and ligands expressed in more than 10% of the cells in the specific cluster were considered in this study.

### 4.6 Cell culture and quantitative PCR

Culture of human HSCs LX‐2 and TGFβ1‐induced activation according to our previous study.^71^ Total RNA was extracted from LX‐2 cells using TRIzol reagent (15596026; Invitrogen). cDNA was synthesized using a reverse transcription kit (FSQ‐301; Toyobo). RNA was amplified using the primers indicated in Table S1. Quantitative RT‐PCR gene expression analysis was performed as our previously described.^71^ The mRNA expression levels of *αSMA* *(Acta2)*, *Pdgfrβ (Pdgfrb)*, *Fn1*, *MCP1 (Ccl2)*, *Il‐1α*, *Il‐6*, *iNOS (Nos2)*, *Cd80*, *Tn* *f*‐*α* *(* *Tnf* *)*, and *Thbs1* were assessed by quantitative PCR (qPCR).

### 4.7 HSC isolation and immunofluorescence

Isolation and culture of mouse primary HSCs from C57BL/6 male mice (6–8 weeks old) were performed as previously described.^72^, ^73^ Briefly, the NPCs suspensions were first collected for further HSC isolation. The suspensions was centrifuged at 400×*g* for 10 min (4°C) to collect pellet. The collected pellet were resuspended in 15% OptiPrep (07820; Stemcell Technologies) and transferred to a 15 mL centrifuge tube after sufficient mixing. Then, 5 mL of 11.5% OptiPrep and 2 mL of Gey's Balanced Salt Solution B^74^ (GBSS‐B) were individually layered carefully onto the cell suspension. The tube was centrifuged at 1400 ×g for 20 min (4°C, low acceleration), and the cell layer between the 11.5% OptiPrep and GBSS‐B was carefully collected. The obtained HSCs were concentrated in the interphase as a white, flocculent structure. After harvesting and washing with GBSS‐B, the HSCs were seeded on uncoated plastic tissue culture dishes in DMEM supplemented with 10% FBS, 4 mM l‐glutamine, 100 U/mL penicillin, and 100 mg/mL streptomycin. The purity of isolated HSC at one and three days following incubation was distinguished under the light microscope by the lipid vesicles they contained and the unique star‐like morphology.^74^ HSCs were employed in assays 1−2 days after seeding, with the first medium change occurring 24 h after seeding. HSCs were treated for 36 h with Tgfβ1 (5 ng/mL, 100−21; PeproTech) and Thbs1 (10 μM, 3074‐TH‐050; R&D Systems) alone, as well as Thbs1 and rapamycin (0.1 μM, S1842; Beyotime) in combination, and then were incubated with the antibodies Pdgfrβ (ab32570; Abcam), Sdc4 (ab24511; Abcam), and αSMA (NBP2‐22120; Novus Biologicals) for the immunofluorescence staining experiment. The fluorescent secondary antibodies were conjugated with Alexa Fluor 488 and 546 (A11008, A11003; Invitrogen). All samples were examined using a fluorescence microscope (Nikon; Eclipse Ts2).

### 4.8 Macrophage and HSC coculture

Transwell inserts (0.4 μm polyester membrane; 3470; Costar) were placed above adherent HSC for macrophage coculture. The iBMDMs were cultured in DMEM supplemented with 10% FBS and 1% penicillin/streptomycin. Knockdown of Thbs1 in iBMDMs was performed by transient transfection of siRNA duplexs or negative control for 24 h, and subsequently, these siRNA‐exposed iBMDMs were stimulated with 50 ng/mL murine IFN‐γ (315‐05; PeproTech) and 100 ng/mL LPS (L3129; Sigma) for 24 h to polarized phenotype.^75^ The knockdown efficacy of siRNA and the mRNA expression of macrophage polarization markers were assessed by qPCR and western blotting. Thbs1 expression in polarized iBMDMs was determined using western blotting and qPCR. The cells were then digested and resuspended in HSC media containing 2% FBS at 400,000 cells/mL and ∼200,000 cells were added to the top of the transwell insert. Coculture proceeded for 48 h and HSC were harvested for RNA and immunofluorescence identification of HSC activation markers.

### 4.9 Immunohistology

Immunohistology was performed on formalin‐fixed and paraffin‐embedded mouse liver sections for αSMA (NBP1‐97722; Novus Biologicals; ab32575; Abcam), Col1a1 (ab34710; Abcam), Pdgfrβ (ab32570; Abcam), Sdc4 (ab24511; Abcam), Thbs1 (ab1823; Abcam), F4/80 (70076s; CST), and CD68 (ab125212; Abcam). Triple and double immunofluorescence staining using a panovue multicolor fluorescent staining kit according to the manufacturer's protocols (10079100020; Panovue). Images were acquired with a High‐content screening system (YOKOGAWA, CQ1) and a fluorescence imaging system (Nikon; Eclipse Ts2).

### 4.10 Western blotting assay

Cells were lysed on ice with lysis buffer (50 mM Tris–HCl, 150 mM NaCl, 1% NP‐40, 0.5% sodium deoxycholate, and 0.1% SDS, pH 7.5) in the presence of a protease inhibitor mixture and phosphatase inhibitors (11697498001; Roche). Protein concentrations were measured using the BCA protein assay kit (23227; Thermo Fisher Scientific). The lysates were separated by SDS‐PAGE and transferred to a PVDF membrane, followed by immunoblotting with Pdgfrβ (ab32570; Abcam), Akt (9272S; CST), p‐Akt (4060S; CST), mTOR (2972S; CST), PI3K‐p110α (4249T; CST), PI3K‐p85 (4257T; CST), p‐PI3K‐p85 (4228T; CST), Thbs1 (ab1823; Abcam) antibodies, and secondary antibodies.

### 4.11 Statistical analysis

The analysis of experimental data and the production of statistical figures were carried out using GraphPad Prism 8.0.1 software. All data are presented as the mean ± standard deviation. The differences in data were analyzed with an unpaired two‐sided Student's *t*‐test or one‐way ANOVA (nonparametric or mixed) for comparisons. A *p* value < 0.05 was considered significant (*p < 0.05, **p < 0.01, ***p < 0.001, ****p < 0.0001, ^#^ *p* < 0.05, ^##^ *p* < 0.01, ^###^ *p* < 0.001, ^####^ *p* < 0.0001).

## AUTHOR CONTRIBUTION

B. H., S. W. H., and X. B. S. conceived and designed the research. B. H. and X. B. S. interpreted the data and wrote the manuscript. K. T. and Y. L. W. collected the clinical samples. B. H., S. C., Y. H. Z., M. Z., S. H. B., and Y. S. collected the data and performed all analyses. S. W. H., J. X. W., Y. Z. L., K. Y. H., and P. S. reviewed and edited the manuscript. All authors contributed to the article and approved the final version of the manuscript.

## CONFLICT OF INTEREST STATEMENT

The authors have declared that no conflict of interest exists.

l

## ETHICS STATEMENT

All investigations involving animals were carried out in line with ethical standards and according to the approved protocol by Shanghai Jiao Tong University (approval number 2021073003). The study involving human participants was reviewed and approved by the ethics board of Shanghai Tongren Hospital (approval number 2020‐035‐01). Written informed consent for research purposes was obtained from all participants.

## Supporting information

Supporting Information

## Data Availability

The raw scRNA‐seq data in this study have been deposited to Genome Sequence Archive (GSA) in BIG Data Center, Beijing Institute of Genomics (BIG) under accession number CRA007803.
