# Supplementary material for: Single‐cell RNA sequencing reveals the heterogeneity and intercellular communication of hepatic stellate cells and macrophages during liver fibrosis
Source: MedComm (2020). 2023 Sep 17;4(5):e378. doi: 10.1002/mco2.378 (PMC10505372; doi:10.1002/mco2.378)
Supplement: Supplementary file 1 — Supporting Information [file MCO2-4-e378-s001.docx]

**Single-cell RNA sequencing reveals the heterogeneity and intercellular communication of hepatic stellate cells and macrophages during liver fibrosis**

Sheng Cheng^1,2#^, Yunhan Zou^3#^, Man Zhang^4#^, Shihao Bai^4^, Kun Tao^5^, Jiaoxiang Wu^2^, Yi Shi^6,7^, Yuelan Wu^2^, Yinzhong Lu^2,8^, Kunyan He^4^, Peng Sun^1^, Xianbin Su^4,7*^, Shangwei Hou^8*^, Bo Han^1,2*^

1. *Department of General Surgery, Tongren Hospital, Shanghai Jiao Tong University School of Medicine, Shanghai, China;*
2. *Key Laboratory for Translational Research and Innovative Therapeutics of Gastrointestinal Oncology, Hongqiao International Institute of Medicine, Tongren Hospital, Shanghai Jiao Tong University School of Medicine, Shanghai, China;*
3. *Department of Biochemistry and Molecular Cell Biology, Shanghai Key Laboratory for Tumor Microenvironment and Inflammation, Shanghai Jiao Tong University School of Medicine, Shanghai, China;*
4. *Key Laboratory of Systems Biomedicine (Ministry of Education), Shanghai Center for Systems Biomedicine, Shanghai Jiao Tong University, Shanghai, China;*
5. *Department of Pathology, Tongren Hospital, Shanghai Jiaotong University School of Medicine, Shanghai, China;*
6. *Bio-X Institutes, Key Laboratory for the Genetics of Developmental and Neuropsychiatric Disorders, Shanghai Jiao Tong University, Shanghai, China;*
7. *eHealth Program of Shanghai Anti-doping Laboratory, Shanghai University of Sport, Shanghai, China;*
8. *Department of Anesthesiology, Tongren Hospital, Shanghai Jiao Tong University School of Medicine, Shanghai, China*

***Correspondence**

Bo Han, Department of General Surgery, Tongren Hospital, Shanghai Jiao Tong University School of Medicine, Shanghai, 200336, China. Email: bhan@shsmu.edu.cn

Shangwei Hou, Department of Anesthesiology, Tongren Hospital, Shanghai Jiao Tong University School of Medicine, Shanghai, 200236, China. Email: housw@sjtu.edu.cn

Xianbin Su, Key Laboratory of Systems Biomedicine (Ministry of Education), Shanghai Center for Systems Biomedicine, Shanghai Jiao Tong University, Shanghai, 200240, China. Email: xbsu@sjtu.edu.cn.

#These authors have contributed equally to this work and share first authorship.

**Table S1. List primers for qPCR.**

| Gene | Forward primer (5’-3’) | Reverse primer (5’-3’) |
| --- | --- | --- |
| **Mouse** |  |  |
| Acta2 | GTCCCAGACATCAGGGAGTAA | TCGGATACTTCAGCGTCAGGA |
| Pdgfrb | TTCCAGGAGTGATACCAGCTT | AGGGGGCGTGATGACTAGG |
| Fn1 | GCAGTGACCACCATTCCTG | GGTAGCCAGTGAGCTGAACAC |
| Cd80 | ACCCCCAACATAACTGAGTCT | TTCCAACCAAGAGAAGCGAGG |
| Il6 | CCAAGAGGTGAGTGCTTCCC | CTGTTGTTCAGACTCTCTCCCT |
| Il1a | CGAAGACTACAGTTCTGCCATT | GACGTTTCAGAGGTTCTCAGAG |
| Nos2 | GTTCTCAGCCCAACAATACAAGA | GTGGACGGGTCGATGTCAC |
| Tnf | CCCTCACACTCAGATCATCTTCT | GCTACGACGTGGGCTACAG |
| Ccl2 | TTAAAAACCTGGATCGGAACCAA | GCATTAGCTTCAGATTTACGGGT |
| Thbs1 | GGGGAGATAACGGTGTGTTTG | CGGGGATCAGGTTGGCATT |
| **Human** |  |  |
| Acta2 | GTGTTGCCCCTGAAGAGCAT | GCTGGGACATTGAAAGTCTCA |
| Pdgfrb | AGACACGGGAGAATACTTTTGC | AGTTCCTCGGCATCATTAGGG |
| Fn1 | AGGAAGCCGAGGTTTTAACTG | AGGACGCTCATAAGTGTCACC |
| Ccl2 | CAGCCAGATGCAATCAATGCC | TGGAATCCTGAACCCACTTCT |


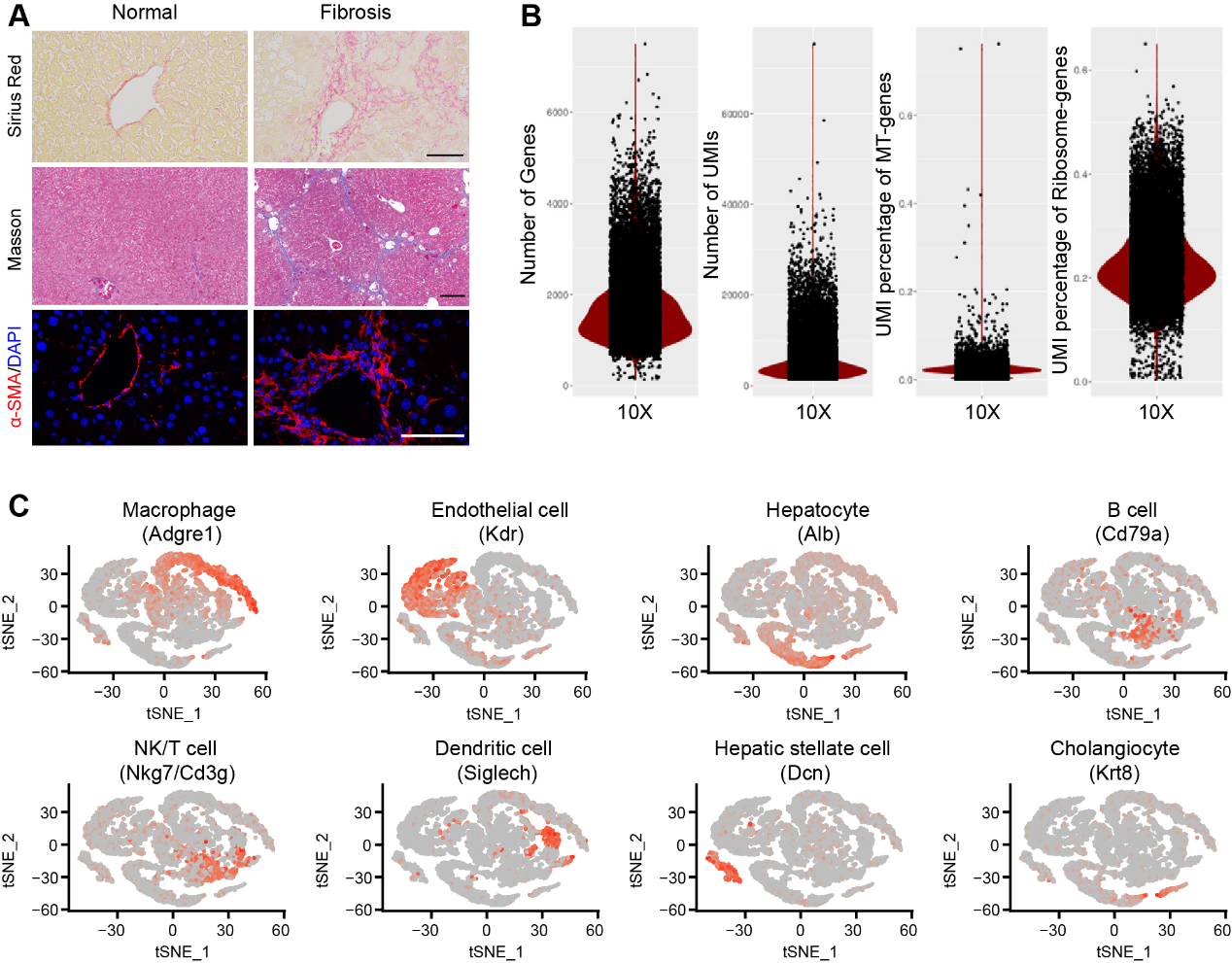


Figure S1: Quality control and annotation of mouse NPCs, related to Figure 1. (A) Representative Sirius Red, Masson and anti-αSMA immunofluorescence staining of liver sections from normal and fibrotic mouse (scale bar, 50 μm). (B) Parameters of mouse NPC single cells after the quality filter. (C) t-SNE plot colored for expression of marker genes of all clusters.


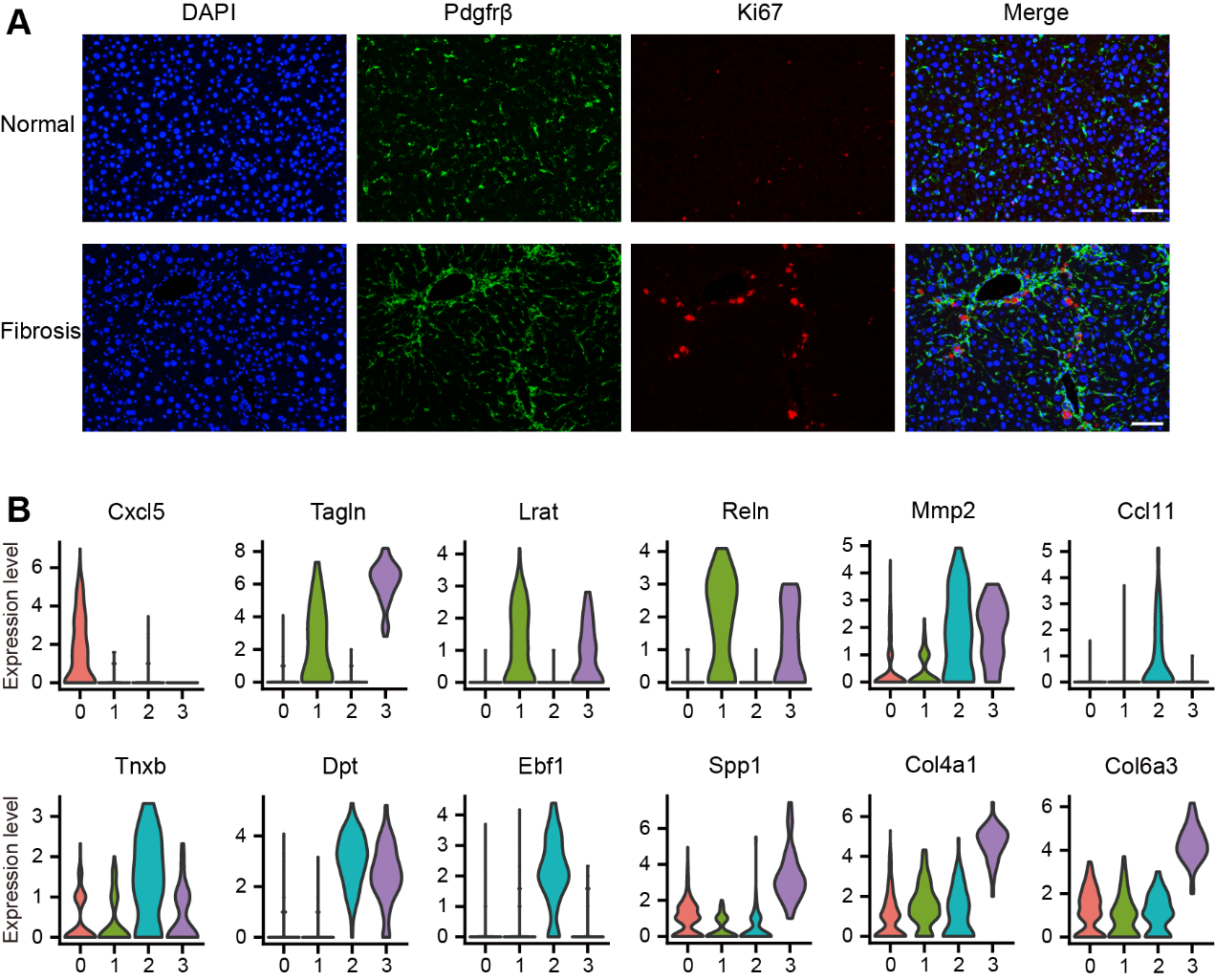


Figure S2: Annotation of mouse hepatic stellate cells, related to Figure 2. (A) Immunofluorescence co-staining for Pdgfrβ and Ki67 in normal and fibrotic mouse livers to identify the proliferation of HSCs under fibrosis (scale bar, 50 μm). (B) Violin plots showing the selected HSC marker and functional genes in four sub-clusters.


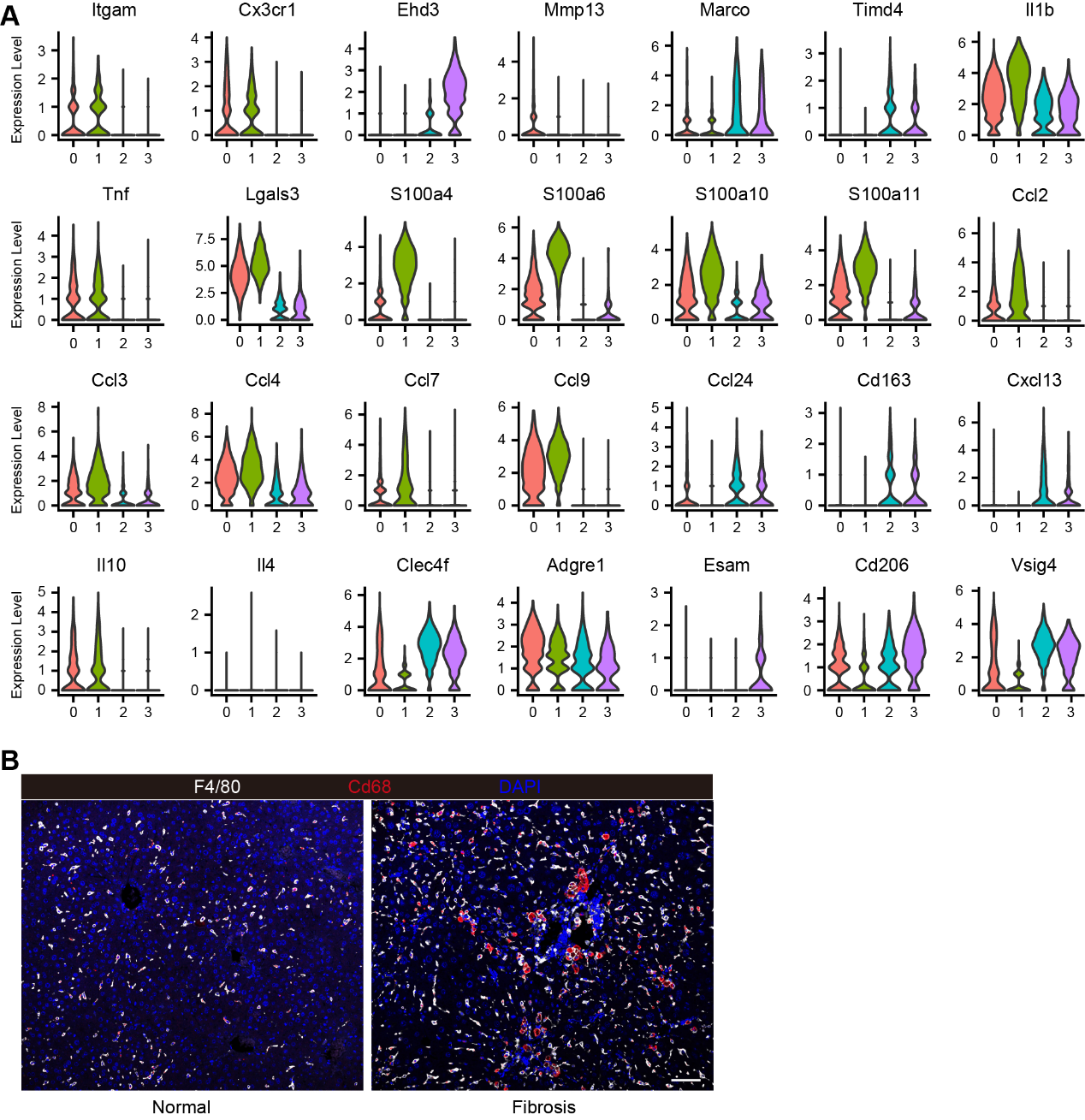


Figure S3: Expression of marker and functional genes of sub-clusters in macrophages, related to Figure 4. (A) Violin plots showing the selected macrophage markers and functional genes in four sub-clusters. (B) Immunofluorescence co-staining for F4/80 and Cd68 in normal and fibrotic mouse livers (scale bar, 25 μm).


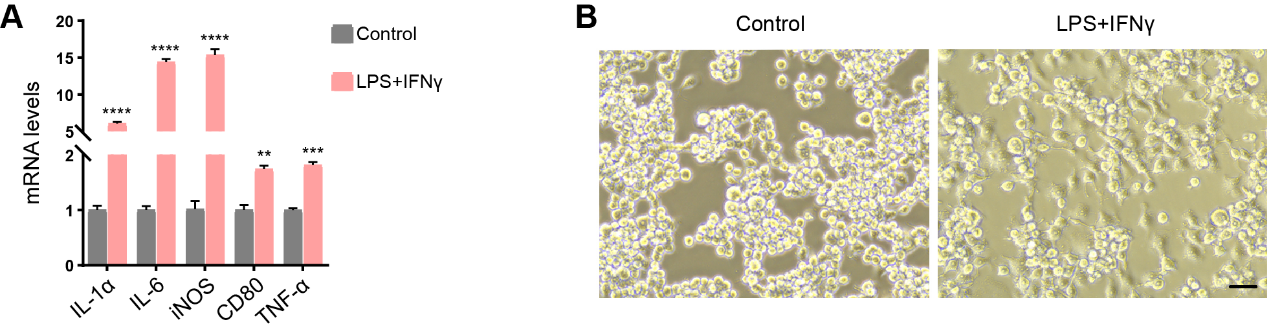


Figure S4: Co-culture analysis of iBMDMs and HSCs, related to Figure 5. (A) mRNA expression level of pro-inflammatory polarized macrophage markers. (B) Morphological changes in iBMDMs activated by LPS and IFN-γ treatment (scale bar, 50 μm).


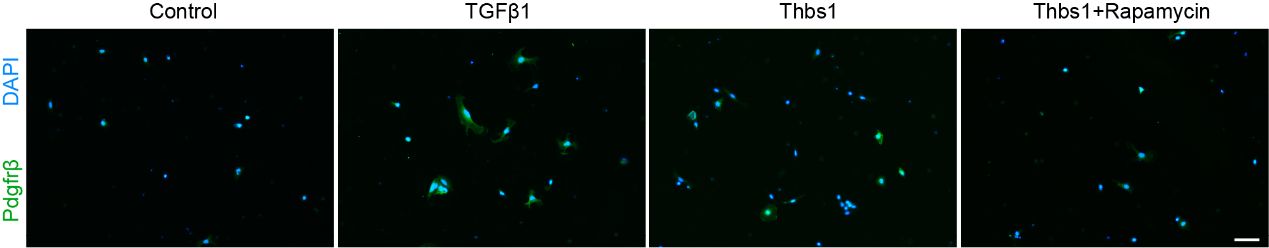


Figure S5: Immunofluorescence staining for Pdgfrβ in quiescent and activated mouse primary HSCs subjected to the indicated treatments (scale bar, 100 μm), related to Figure 6.
